# *In Silico* and *In Vitro* Analysis of Bacoside A Aglycones and Its Derivatives as the Constituents Responsible for the Cognitive Effects of *Bacopa monnieri*

**DOI:** 10.1371/journal.pone.0126565

**Published:** 2015-05-12

**Authors:** Seetha Ramasamy, Sek Peng Chin, Sri Devi Sukumaran, Michael James Christopher Buckle, Lik Voon Kiew, Lip Yong Chung

**Affiliations:** 1 Department of Pharmacy, Faculty of Medicine, University of Malaya, Kuala Lumpur, Malaysia; 2 Department of Pharmacology, Faculty of Medicine, University of Malaya, Kuala Lumpur, Malaysia; 3 Center for Natural Products and Drug Research (CENAR), University of Malaya, Kuala Lumpur, Malaysia; Rutgers University, UNITED STATES

## Abstract

*Bacopa monnieri* has been used in Ayurvedic medicine to improve memory and cognition. The active constituent responsible for its pharmacological effects is bacoside A, a mixture of dammarane-type triterpenoid saponins containing sugar chains linked to a steroid aglycone skeleton. Triterpenoid saponins have been reported to be transformed *in vivo* to metabolites that give better biological activity and pharmacokinetic characteristics. Thus, the activities of the parent compounds (bacosides), aglycones (jujubogenin and pseudojujubogenin) and their derivatives (ebelin lactone and bacogenin A1) were compared using a combination of *in silico* and *in vitro* screening methods. The compounds were docked into 5-HT_1A_, 5-HT_2A_, D_1_, D_2_, M_1_ receptors and acetylcholinesterase (AChE) using AutoDock and their central nervous system (CNS) drug-like properties were determined using Discovery Studio molecular properties and ADMET descriptors. The compounds were screened *in vitro* using radioligand receptor binding and AChE inhibition assays. *In silico* studies showed that the parent bacosides were not able to dock into the chosen CNS targets and had poor molecular properties as a CNS drug. In contrast, the aglycones and their derivatives showed better binding affinity and good CNS drug-like properties, were well absorbed through the intestines and had good blood brain barrier (BBB) penetration. Among the compounds tested *in vitro*, ebelin lactone showed binding affinity towards M_1_ (K_i_ = 0.45 μM) and 5-HT_2A_ (4.21 μM) receptors. Bacoside A and bacopaside X (9.06 μM) showed binding affinity towards the D_1_ receptor. None of the compounds showed any inhibitory activity against AChE. Since the stimulation of M_1_ and 5-HT_2A_ receptors has been implicated in memory and cognition and ebelin lactone was shown to have the strongest binding energy, highest BBB penetration and binding affinity towards M_1_ and 5-HT_2A_ receptors, we suggest that *B*. *monnieri* constituents may be transformed *in vivo* to the active form before exerting their pharmacological activity.

## Introduction

*Bacopa monnieri* (Linn.) Pennell (Scrophulariaceae), also known as Brahmi, is a reputed Ayurvedic herb noted to improve memory and cognition [1]. These traditional claims have been supported by extensive *in vitro*, *in vivo* and clinical studies conducted over the last two decades using the plant extract and its constituents [2]. A meta-analysis of randomized controlled trials on the cognitive effects of *B*. *monnieri* extract also suggests that *B*. *monnieri* has the potential to improve cognition [3]. Other important pharmacological activities shown by *B*. *monnieri* include antiepileptic, anxiolytic, antidepressant, sedative, antioxidant and anti-inflammatory activities [4]. Various mechanisms of action for its cognitive effects have been proposed including acetylcholinesterase (AChE) inhibition, β-amyloid reduction, antioxidant neuroprotection, neurotransmitter modulation (acetylcholine [ACh], 5-hydroxytryptamine [5-HT], dopamine [DA]), choline acetyltransferase activation and increased cerebral blood flow [5].

**Fig 1:**
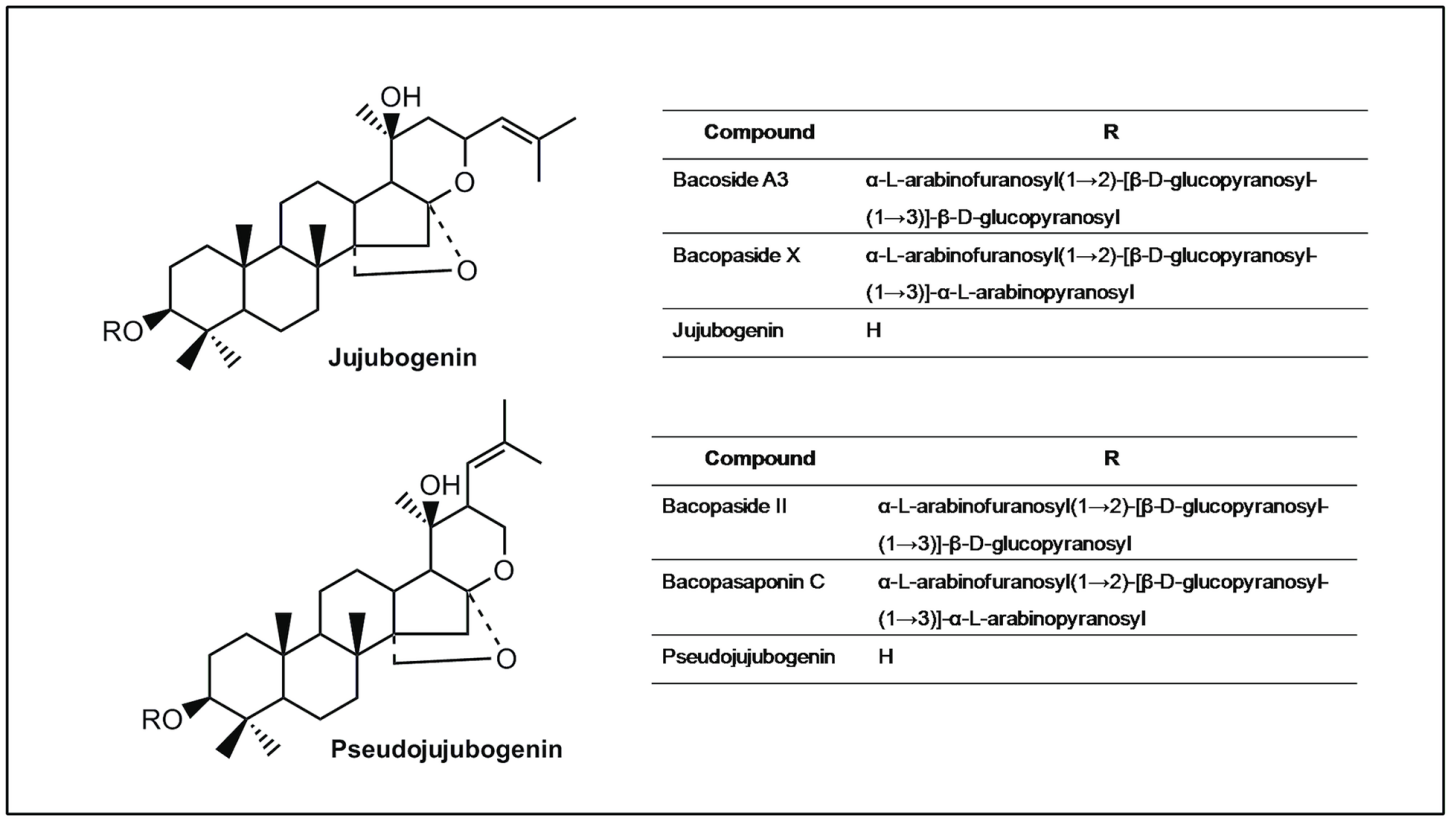
Structures of bacoside A saponin glycosides and aglycones. Bacoside A is a mixture of bacoside A3, bacopaside II, bacopaside X and bacopasaponin C. These bacosides are dammarane-type triterpenoid saponins that have three sugar chains linked to a nonpolar triterpene aglycone skeleton.

Characteristic saponins called ‘bacosides’, especially bacoside A, have been considered to be the main bioactive constituents responsible for the cognitive effects of *B*. *monnieri* [6–8]. Bacoside A is a mixture of four triglycosidic saponins, namely bacoside A3, bacopaside II, bacopasaponin C and the jujubogenin isomer of bacosaponin C (bacopaside X) [9]. These bacosides are dammarane types of triterpenoid saponins with jujubogenin or pseudojujubogenin moieties as the aglycone units (Fig 1) [10].

**Fig 2:**
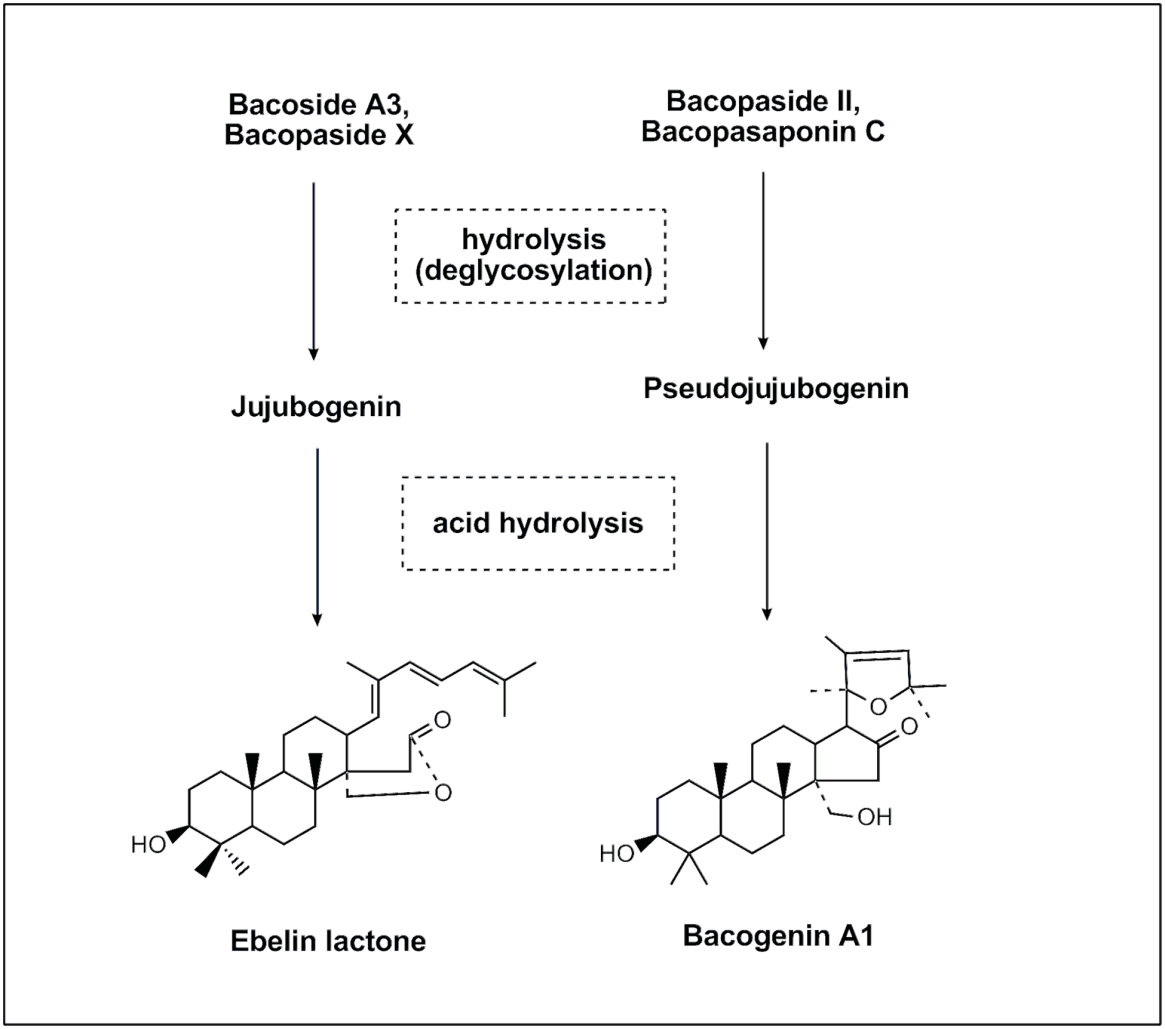
Formation of ebelin lactone and bacogenin A1. Bacoside A components form aglycone jujubogenin and pseudojujubogenin through deglycosylation and further acid hydrolysis yields ebelin lactone and bacogenin A1.

Saponins are susceptible to glycosidic cleavage at various sites to form secondary metabolites and finally the aglycone [11]. Triterpenoid saponins from other neuropharmacologically active plants such as ginsenoside [12] and jujuboside [13, 14] have shown that instead of the parent saponins, the metabolites transformed *in vivo* give better biological activity and pharmacokinetic characteristics. A recent study by Le et al. [15] showed that a *B*. *monnieri* extract had a negligible effect on the *in vitro* activity of AChE of brain tissues, whereas its daily systemic administration reduced the *ex vivo* activity of AChE in brain tissues. The study proposed that chemical constituent(s) of *B*. *monnieri* may be converted to their active form(s) *in vivo* with the ability to inhibit the activity of AChE in the brain. A recent report shows *B*. *monnieri* extracts inhibit some human cytochrome P450 (CYP) drug metabolizing enzymes [16]. It can also alter the expression of rat hepatic and intestinal CYP drug metabolizing enzymes and intestinal P-glycoprotein [17]. Thus, it is conceivable that the bacoside constituents present in *B*. *monnieri* extracts may be metabolized *in vivo* to active forms before exerting their pharmacological activities. Through sequential deglycosylation, bacoside A3, bacopaside II, bacopaside X and bacopasaponin C can be transformed to their aglycones jujubogenin or pseudojujubogenin. Jujubogenin and pseudojujubogenin can be further acid hydrolyzed to form ebelin lactone and bacogenin A1, respectively (Fig 2) [18].

Therefore, in this study, we aim to compare the activity of the parent compounds (bacosides) with the aglycones (jujubogenin and pseudojujubogenin) and their derivatives (ebelin lactone and bacogenin A1) by studying the central nervous system (CNS) receptor (muscarinic, serotonin and dopamine) binding and AChE inhibition activities using a combination of *in silico* and *in vitro* screening methods. These assays were chosen because most CNS-related disorders such as schizophrenia, Alzheimer’s disease, epilepsy, and Parkinson's disease are related to neurotransmitters such as acetylcholine, dopamine, 5-hydroxytryptamine (5-HT) and their receptors [19]. In addition, physicochemical and ADMET (Absorption, Distribution, Metabolism, Excretion and Toxicity) properties of the compounds were calculated using web-based applications and software to predict whether the compounds are orally active and have CNS drug properties. To the best of our knowledge, this is the first study on bacoside A aglycone and its derivatives.

## Materials and Methods

### Chemicals and reagents

Recombinant human membrane preparations for M_1_ (expressed in CHO cells; Lot no. 1706299), 5-HT_1A_ (HEK293 cells; no. 1812363), 5-HT_2A_ (CHO cells; no. 1813092), D_1_ (L cells; no. 1840059), and D_2S_ (CHO cells; no. 1820984) receptors and MicroScint-O scintillation cocktail were procured from Perkin Elmer (Waltham, MA, USA). [^3^H] *N*-methylscopolamine (NMS), [^3^H] 8-OH-DPAT, [^3^H] ketanserin, [^3^H] SCH 23390 and [^3^H] methylspiperone were purchased from American Radiolabeled Chemicals (St. Louis, MO, USA). Atropine, 5-carboxamidotryptamine (5-CT), ketanserin, SCH 23390 hydrochloride, haloperidol, serotonin hydrochloride, mianserin, human recombinant acetylcholinesterase expressed in HEK 293 cells, acetylthiocholine iodide (ATChI), 5, 5’-dithiobis [2-nitrobenzoic acid] (DTNB) and donepezil hydrochloride were obtained from Sigma-Aldrich (St. Louis, MO, USA). Bacoside A, bacoside A3, bacopaside II, bacopaside X and bacopasaponin C were purchased from Chromadex Inc. (Irvine, CA, USA). Jujubogenin and ebelin lactone were purchased from Shanghai IS Chemical Technology Ltd. (Jinshan, Shanghai, China). Unless stated otherwise, all other reagents of analytical grade were obtained through standard commercial sources.

### *In silico* studies

#### Molecular docking

The two-dimensional (2-D) structures of the ligands (compounds) were built using ChemBioDraw Ultra 11.0 (Perkin Elmer) and converted to 3-dimensional (3-D) structures using Chem3D Pro 12.0 (Perkin Elmer). The resulting structures were subjected to energy minimization by MM2 force field and saved as MOL files. Finally the pdbqt formats (the input format of the docking software) of the ligands were prepared with AutoDockTools 1.5.6 [20] using default parameters.

For AChE, crystal structures of human AChE (hAChE) in complex with donepezil (2.35 Å) (PDB ID: 4EY7) and hAChE in complex with fasciculin II (2.76 Å) (PDB ID: 1B41) were extracted from the protein data bank (PDB) as a pdb file. The heteroatoms and water molecules were removed using Discovery Studio Visualizer 3.1 (Accelrys, San Diego, CA, USA). Hydrogens were added and double coordinates were corrected using HyperChem Pro 6.0 (Hypercube Inc., Gainesville, FL, USA). Then, hydrogens were added again, non-polar hydrogens were merged and the missing atoms were repaired using AutoDockTools 1.5.6. Finally, Kollman charges were added and AutoDock 4 type atoms were assigned to the protein. For the CNS receptors [serotonin: 5-HT_1A_, 5-HT_2A_; dopamine: D_1_, D_2_ and muscarinic acetylcholine (mACh): M_1_], validated homology models built from the previous work of our group were used [21, 22].

Docking studies were performed with AutoDock 4.2 [20], using a Lamarckian genetic algorithm [23] with a flexible ligand and a rigid receptor, a population size of 300, a maximum of 250,000 generations and 2,500,000 evaluations for 100 GA runs. The root mean square deviation (RMSD) tolerance was set to 2.0 Å for the clustering of docked results. Docking grids and the grid box was set to cover the transmembrane (TM) domain (for CNS receptors) and entire protein (for AChE enzyme). Ligand-receptor interactions were viewed using Discovery Studio Visualizer 3.1. Maestro 9.2 and PyMOL 1.3 (Schrödinger, LLC, New York, USA) were used to produce 2-D and 3-D figures.

### Drug-like properties

Discovery Studio 4.0 molecular properties and ADMET descriptors were used to determine the CNS drug-like properties of the compounds. The ADMET descriptor estimates a range of properties for the compounds using QSAR models. Since these compounds are taken orally and were screened for CNS activity, intestinal absorption properties and blood brain barrier (BBB) penetration were evaluated. The molecular properties include molecular weight, polar surface area (PSA), log *P* (octanol-water partition coefficient), H-bond donors, H-bond acceptors and number of rotatable bonds.

### *In vitro* radioligand receptor binding assay

The assay was performed according to the methods published previously [24, 25]. Bacoside A, bacopasaponin C, bacopaside X and bacoside A3 were assayed up to 100 μM whereas bacopaside II, jujubogenin and ebelin lactone were only assayed up to 30 μM due to poor solubility. Pseudojujubogenin and bacogenin A1 were not available for purchase at the time when the work was carried out. All compounds were dissolved in dimethylsulfoxide (DMSO) and the solvent was kept below 1% (v/v) in the final reaction mixture. Briefly, in each well, 100 μl of the respective membrane preparations (μg/well), 50 μl of the respective [^3^H]-ligand and 50 μl of the test compounds were added and the total assay reaction volume was made up to 200 μl by adding assay buffer. In place of the test compounds, 50 μl of 4x concentration of atropine (M_1_), serotonin (5-HT_1A_), mianserin (5-HT_2A_) and haloperidol (D_1_, D_2_) was added to respective wells to determine the non-specific binding (NSB) (or 100% inhibition) whereas 50 μl of assay buffer was added to determine the total binding (TB) of radioligand (or 0% inhibition). The reaction mixture was incubated for 60 or 120 min at room temperature or 37°C for the respective membranes. The reaction was stopped by rapid filtration onto GF/C filter plates (presoaked in 0.5% polyethyleneimine) using FilterMate (Perkin Elmer) cell harvester and washed with wash buffer (200 μl for 5 times) to remove unbound ligands. The filter plates were dried at 60°C for 15 minutes before the application of Bottom Seal and addition of 50 μl MicroScint-O scintillation cocktail. The top of the plate was then sealed with TopSeal A. Radioactivity (CPM) was counted using TopCount NXT microplate scintillation counter (Perkin-Elmer). A summary of the reaction components and the assay conditions are listed in Table 1. The data were analyzed by non-linear regression using Prism Version 5.0 (GraphPad Inc., San Diego CA, USA). The percentage of specific binding of radioligand in the presence of test compounds was calculated using the standard data reduction algorithm: ([B-NSB] / [TB-NSB]) × 100) where B is the binding in the presence of test compounds, NSB is the non-specific binding in the presence of excess reference ligand and TB is the total binding. *K*_i_ values were calculated from the IC_50_ values using the Cheng-Prusoff equation [26].

**Table 1:** Summary of radioligand receptor binding assay components and reactions according to each receptor.

Assay Components	Muscarinic (M_1_)	Serotonin (5-HT_1A_)	Serotonin (5-HT_2A_)	Dopamine (D_1_)	Dopamine (D_2s_)
**Assay Buffer**	PBS pH 7.4	50 mM Tris-HCl pH 7.4, 5 mM MgSO_4_	50 mM Tris-HCl pH 7.4, 4 mM CaCl_2_, 0.1% Ascorbic acid	50 mM Tris-HCl pH 7.4, 1.5 mM CaCl_2_, 5 mM MgCl_2_, 5 mM EDTA, 5 mM KCl	50 mM Tris-HCl pH 7.4, 120 mM NaCl, 5 mM KCl, 5 mM MgCl_2_, 1 mM EDTA
**[** ^**3**^ **H] Ligand, nM**	0.1 nM ([^3^H]-NMS)	2 nM ([^3^H]-8-OH-DPAT)	1 nM ([^3^H]-Ketanserin)	0.2 nM ([^3^H]-SCH 23390)	0.2 nM ([^3^H]-Methylspiperone)
**NSB Ligand**	Atropine (10 μM)	Serotonin (10 μM)	Mianserin (20 μM)	Haloperidol (20 μM)	Haloperidol (10 μM)
**Human Recombinantmembranes**	17.5 μg/well	16 μg/well	5 μg/well	2 μg/well	3 μg/well
**Incubation**	120 min @ 27°C	120 min @ 37°C	60 min @ 27°C	60 min @ 27°C	120 min @ 27°C
**Wash Buffer**	50 mM Tris-HCl pH 7.4, 154 mM NaCl	50 mM Tris-HCl pH 7.4	50 mM Tris-HCl pH 7.4	50 mM Tris-HCl pH 7.4	50 mM Tris-HCl pH 7.4, 154 mM NaCl
**Washes (200 μl/well)**	5	5	5	5	5

### *In vitro* AChE inhibition assay

The inhibitory activity of the compounds toward AChE was determined by following the method of Ellman et al. [27] with minor modifications using human recombinant AChE and acetylthiocholine as a substrate. Fifty-microliters (50 μl) of AChE enzyme (0.1 ng/well) in assay buffer [0.1 M sodium phosphate, 0.05% (w/v) Brij35], pH 7.5 and 25 μl of 4× concentrations of the test compounds were mixed in a microplate and left to incubate at room temperature for 30 minutes. Subsequently, 25 μl of a 4× ATChI / DTNB mixture (final concentration 200 μM / 100 μM) was added to the respective wells. This substrate mixture was prepared 5 min prior to being added to the plate in equal volumes of ATChI and DTNB. The hydrolysis of acetylthiocholine was monitored by measuring the absorbance due to yellow 5-thio-2-nitrobenzoate anion in a kinetic mode at a wavelength of 405 nm for 10 min. The enzyme activity was measured in the presence (A_sample_) and in the absence (A_control_) of the test compounds. All the tests were carried out in triplicate and the enzyme inhibition was calculated as: % Inhibition = 100 –[(A_sample_) / (A_control_) x 100].

## Results

### The aglycones show better docking than the parent bacosides

Docking results were analyzed and the best-docked conformation was chosen based on the number of conformations in a cluster and the estimated free energy of binding. Higher numbers of conformations and the lowest binding energy indicate better affinity of the compound to the CNS receptor and AChE enzyme. As shown in Table 2, the docking of the parent compounds, bacopasaponin C, bacopaside X, bacopaside II and bacoside A3 in the CNS receptors and AChE gave a very low number of conformations in a cluster and were not able to fit into the orthosteric site of the CNS receptors and AChE. In contrast, the aglycones (jujubogenin, pseudojujubogenin, ebelin lactone and bacogenin A1) with higher number of conformations in a cluster, docked better than the parent compounds in the CNS receptors and AChE. These results indicate that the binding pockets of the CNS receptors and AChE were not able to accommodate the large glycoside groups on the bacosides. Among these compounds, ebelin lactone showed the strongest binding towards all the CNS receptors, with the lowest estimated free energy of binding.

**Table 2:** Total number of conformations in a cluster and binding energy of compounds for 5-HT_1A_, 5-HT_2A_, D_1_, D_2_ and M_1_ receptors and AChE (in complex with fasciculin or donepezil) enzyme.

Compound	Total number of conformations in a cluster (estimated binding energy kcal/mol)						
	5-HT_1A_	5-HT_2A_	D_1_	D_2_	M_1_	AChE (fasciculin)	AChE (donepezil)
**Bacopa-saponin C**	13 (-9.99)	6 (-12.25)	1 (-11.12)	4 (-13.33)	4 (-12.94)	5 (-8.63)	5 (-10.90)
**Bacopaside X**	7 (-6.97)	6 (-12.25)	2 (-10.50)	1 (-10.64)	1 (-11.68)	3 (-9.61)	5 (-8.35)
**Bacopaside II**	8 (-6.43)	1 (-11.18)	7 (-10.51)	2 (-10.44)	5 (-12.06)	3 (-8.02)	3 (-7.88)
**Bacoside A3**	3 (-6.03)	1 (-11.38)	1 (-9.74)	1 (-9.75)	3 (-12.23)	6 (-8.21)	4 (-7.30)
**Jujubogenin**	**41 (-9.31)**	**25 (-8.54)**	**63 (-8.93)**	**50 (-8.90)**	**70 (-10.68)**	**99 (-11.65)**	**99 (-11.63)**
**Psuedo-jujubogenin**	**79 (-9.42)**	**24 (-8.28)**	**16 (-8.99)**	**63 (-9.26)**	**100 (-11.33)**	**90 (-11.41)**	**96 (-11.40)**
**Ebelin Lactone**	**97 (-9.58)**	**38 (-10.79)**	**28 (-9.27)**	**78 (-11.06)**	**79 (-11.71)**	**83 (-11.59)**	**83 (-11.59)**
**Bacogenin A1**	**49 (-9.07)**	**24 (-9.19)**	**38 (-8.56)**	**28 (-9.52)**	**68 (-10.71)**	**74 (-9.26)**	**65 (-11.59)**

### The aglycones have better CNS drug-like properties than the parent bacosides

Since *B*. *monnieri* is taken orally and has neuropharmacological activities, the active constituents that give the pharmacological activity are necessarily orally and CNS active, i.e., the compound must be absorbed through the intestine and penetrate the BBB. Through various studies, the accepted criteria for CNS drug properties have been found to include molecular weight < 450, polar surface area (PSA) < 60–70 Å^2^ (upper limit is 90 Å^2^), Log *P* < 5, H-bond donor < 3, H-bond acceptor < 7 and number of rotatable bonds < 8 [28]. From Table 3, the parent bacosides (bacopasaponin C, bacopaside X, bacopaside II, bacoside A3) fail to meet all but one (Log *P* < 5) criteria of oral CNS drug candidates. In particular, they fail to meet the criteria for molecular weight (> 899 Da), hydrogen-bonding capacity (hydrogen bond acceptors, 17 to 18; hydrogen bond donors 9 to 10) and molecular flexibility (number of rotatable bonds, 9 to 10). These unfavorable physiochemical traits of the parent bacosides most likely result in poor membrane permeability through the intestine and BBB. However, the aglycones (jujubogenin, pseudojujubogenin, ebelin lactone and bacogenin A1) showed better CNS drug-like properties by meeting four of the required criteria. The removal of the sugar moieties decreases the molecular weight, hydrogen-bonding capacity and molecular flexibility, and increases the lipophilicity of the aglycones (Log *P*, 5.46 to 7.22) compared to the corresponding parent bacosides (Log *P*, 3.30 to 3.72). Although the molecular weight for the aglycones are slightly more than 450, according to Hansch et al. [29] small molecules may undergo significant passive lipid-mediated transport through the BBB when the molecular mass is kept in or below the 400–600 Da range. Furthermore, increasing lipophilicity of aglycones also tends to increase their brain permeation [28].

**Table 3:** The physicochemical properties of bacopasaponin C, bacopaside X, bacopaside II, bacoside A3, jujubogenin, pseudojujubogenin, ebelin lactone and bacogenin A1.

Compounds	Molecular Weight (< 450)	PSA (< 60–70 Å^2^)	Log *P* (< 5)	H-bond donor (< 3)	H-bond acceptor (< 7)	No. of rotatable bonds (< 8)
**Bacopasaponin C**	899.07	256	3.72	9	17	9
**Bacopaside X**	899.07	256	3.54	9	17	9
**Bacopaside II**	929.10	279	3.48	10	18	10
**Bacoside A3**	929.10	279	3.30	10	18	10
**Jujubogenin**	**472.70**	**59**	**7.22**	**2**	**4**	**1**
**Psuedojujubogenin**	**472.70**	**59**	**6.89**	**2**	**4**	**1**
**Ebelin Lactone**	**454.68**	**47**	**6.77**	**1**	**3**	**3**
**Bacogenin A1**	**472.70**	**67**	**5.46**	**2**	**4**	**2**

**Fig 3:**
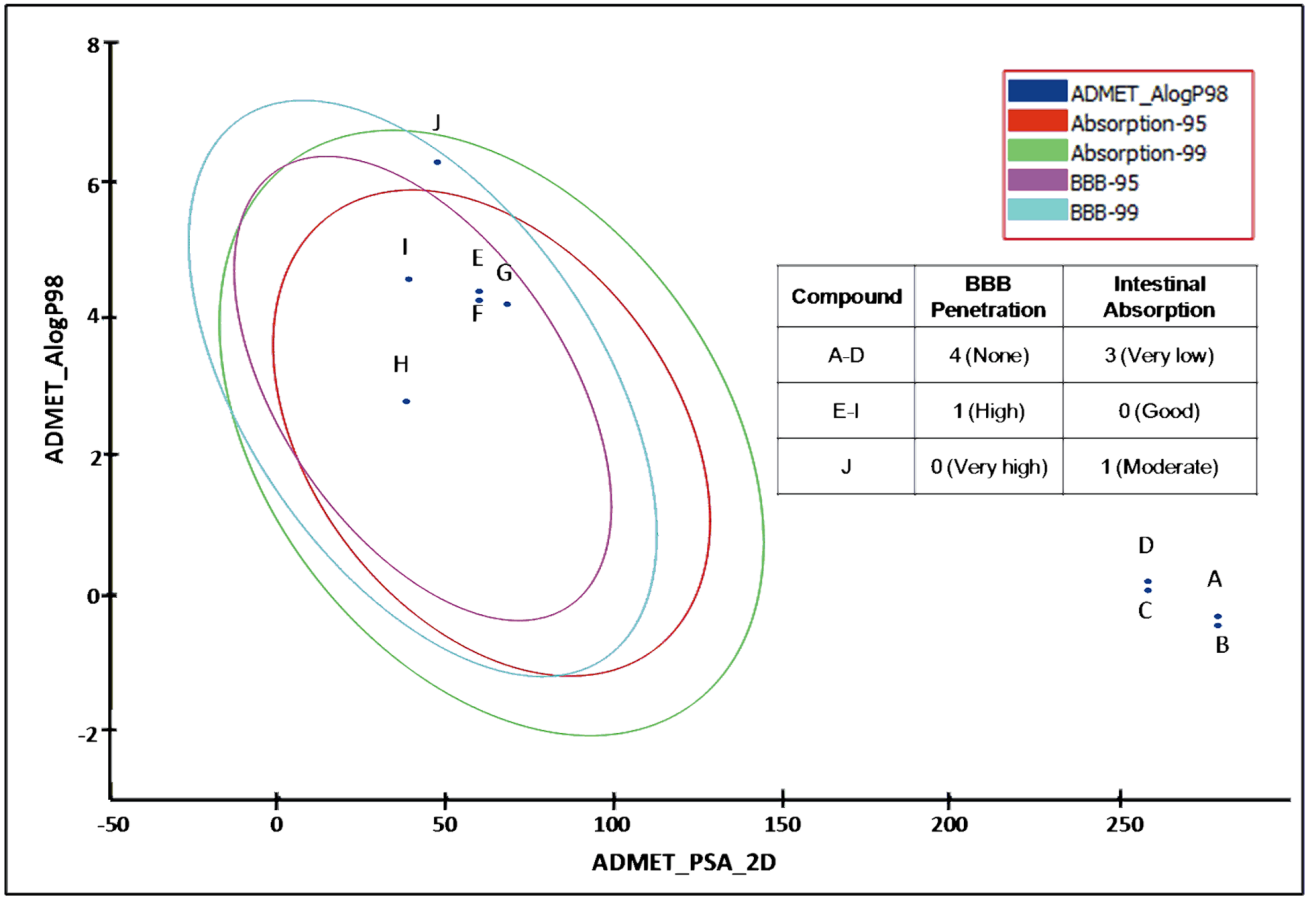
BBB penetration and intestinal absorption properties by ADMET descriptors. (A) bacoside A3, (B) bacopaside II, (C) bacopasaponin C, (D) bacopaside X, (E) jujubogenin, (F) pseudojujubogenin, (G) bacogenin A1, (H) tacrine, (I) donepezil and (J) ebelin lactone. Tacrine and donepezil were used as standard orally active CNS drugs. ADMET prediction level for Human intestinal absorption (HIA)- 0 (good), 1 (moderate), 2 (poor), 3 (very poor); Blood brain barrier (BBB)- 0 (very high penetrant), 1 (high), 2 (medium), 3 (low) and 4 (undefined). The aglycones and its acid hydrolysis derivatives showed better intestinal absorption and BBB penetration compared to the parent bacosides.

Compounds that are taken orally for CNS activity should be able to be absorbed from the intestines and penetrate the BBB. The ADMET human intestinal absorption (HIA) and BBB penetration model are defined by 95% and 99% confidence ellipses in the ADMET_PSA_2D, ADMET_AlogP98 plane (Fig 3) [30, 31]. These ellipses describe the regions where compounds that are both well-absorbed and able to penetrate the BBB are expected to be found. Compounds outside the 95% and 99% confidence ellipsoids are considered to have very low intestinal absorption and BBB penetration. The ADMET descriptor gives four prediction levels within the 95% and 99% confidence ellipsoids for HIA and BBB. The four levels for HIA are 0 (good), 1 (moderate), 2 (poor), 3 (very poor); whereas for BBB, 0 (very high penetrant), 1 (high), 2 (medium), 3 (low) and 4 (undefined). As shown in Fig 3, parent compounds bacopasaponin C, bacopaside X, bacopaside II and bacoside A3 had no BBB penetration and showed very poor intestinal absorption. In contrast, the aglycones jujubogenin, pseudojujubogenin, ebelin lactone and bacogenin A1 were well absorbed through the intestine and had high BBB penetration. Among the compounds ebelin lactone had the highest BBB penetration and predicted to be moderately absorbed through the intestine.

Overall, the *in silico* studies demonstrated that the parent bacosides with glycones attached were not able to dock into CNS receptors and AChE, and had poor molecular and ADMET properties as CNS drugs. On the other hand, the aglycones and their acid hydrolysis derivatives showed better binding affinity towards the CNS receptors and AChE enzyme and conform to the required criteria for intestinal absorption and penetration of the BBB.

### Ebelin lactone interacts with M_1_ and 5-HT_2A_ receptors

At the time of this study, pseudojujubogenin and bacogenin A1 were not available for purchase. Hence, bacopasaponin C, bacopaside X, bacopaside II, bacoside A3, bacoside A (mixture of the four bacosides), jujubogenin and ebelin lactone were further analyzed by *in vitro* assays. The compounds were assayed *in vitro*, for their ability to displace [^3^H] NMS, [^3^H] 8-OH-DPAT, [^3^H] ketanserin, [^3^H] SCH 23390 and [^3^H] methylspiperone from M_1_, 5-HT_1A_, 5-HT_2A_, D_1_, and D_2_ receptors, respectively, and to inhibit AChE activity. The results of the receptor binding assays are shown in Table 4.

**Table 4:** CNS receptor binding affinities of bacosides and aglycones.

Compound^a^	IC_50_ (μM)				
	M_1_	5-HT_1A_	5-HT_2A_	D_1_	D_2_
**Bacoside A**	> 100	> 100	> 100	**24.65 ± 3.76 (*K***_**i**_, **12.14 ± 1.68 μM)**	> 100
**Bacopa-saponin C**	> 100	> 100	> 100	> 100	> 100
**Bacopaside X**	> 100	> 100	> 100	**19.49 ± 3.07 (*K***_**i**_, **9.06 ± 1.36 μM)**	> 100
**Bacoside A3**	> 100	> 100	> 100	> 100	> 100
**Bacopaside II**	> 30	> 30	> 30	> 30	> 30
**Jujubogenin**	> 30	> 30	> 30	> 30	> 30
**Ebelin Lactone**	**0.80 ± 0.19 (*K***_**i**_, **0.45 ± 0.11 μM)**	> 30	**14.48 ± 4.98 (*K***_**i**_, **4.21 ± 1.45 μM)**	> 30	> 30

Values are expressed as the mean ± S.D. of three determinations with two independent experiments.

^a^Bacoside A, bacopasaponin C, bacopaside X and bacoside A3 were assayed up to 100 μM whereas bacopaside II, jujubogenin and ebelin lactone were assayed up to 30 μM due to poor solubility.

Most of the parent bacoside compounds did not show binding affinity towards M_1_, 5-HT_1A_, 5-HT_2A_, D_1_ and D_2_ receptors except bacoside A and bacopaside X, which showed some affinity towards D_1_ receptor. Since bacopaside X is part of the mixture in bacoside A (*K*_i_ = 12.14 μM), the binding might be due to bacopaside X (*K*_i_ = 9.06 μM). Contrary to the *in silico* result, the aglycone jujubogenin did not show binding affinity towards all the receptors tested here. However, its derivative ebelin lactone showed affinity towards M_1_ (*K*_i_ = 0.45 μM) and 5-HT_2A_ (*K*_i_ = 4.21 μM) receptors, which are implicated in memory and learning processes [32, 33]. The *K*_i_ values of ebelin lactone for M_1_ and 5-HT_2A_ are comparable to that of M_1_ agonists acetylcholine and pilocarpine (*K*_i_ = 59 and 2.7 μM, respectively) [25] and 5-HT agonist serotonin (*K*_i_ = 23.9 μM) [34]. However, none of the compounds showed any inhibitory activity against AChE.

### Ebelin lactone may act as an allosteric modulator of M_1_ and 5-HT_2A_ receptors via non-polar interactions

**Fig 4:**
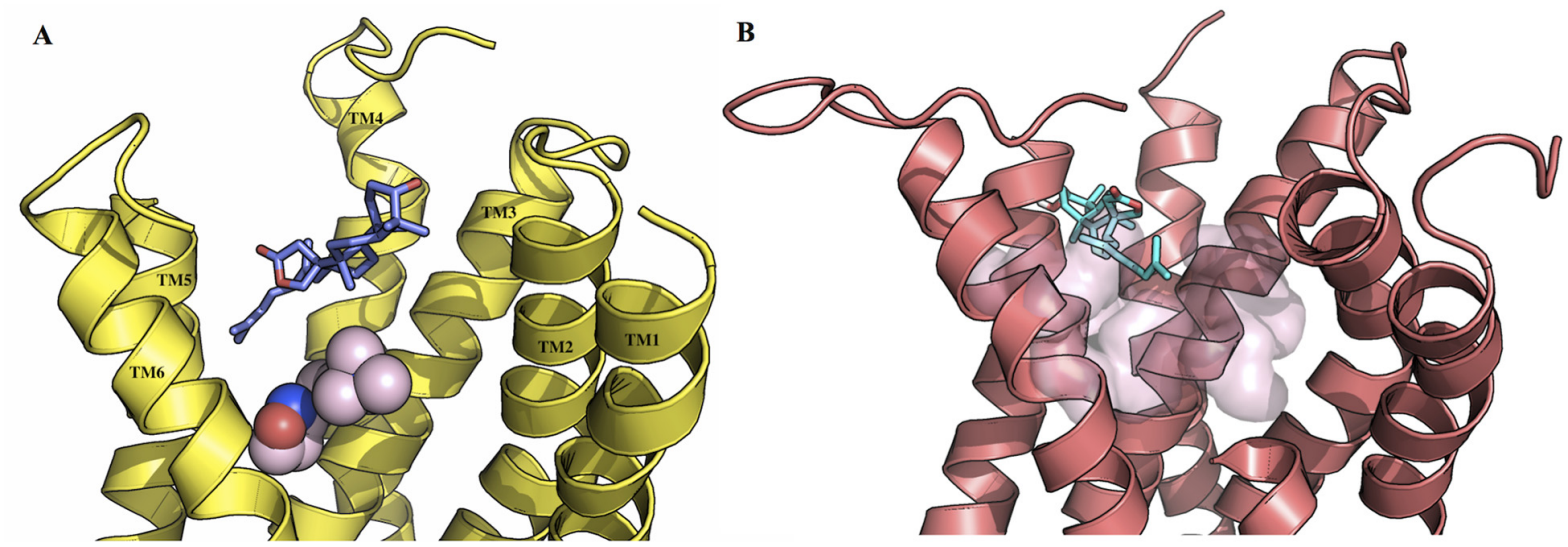
Docking of the ebelin lactone to (A) the M_1_ mAChR and (B) the 5-HT_2A_ models. Iperoxo (sphere) from the crystal structure of M_2_ mAChR (PDB code: 4MQS) was used to show the orthosteric site in the M_1_ mAChR, and the transparent surface represents the orthosteric site of the 5-HT_2A_ receptor. Ebelin lactone bound to a cavity directly above the orthosteric site suggesting it could be an allosteric modulator. For the purpose of clarity, some of the loops and transmembrane helix are not shown.

**Fig 5:**
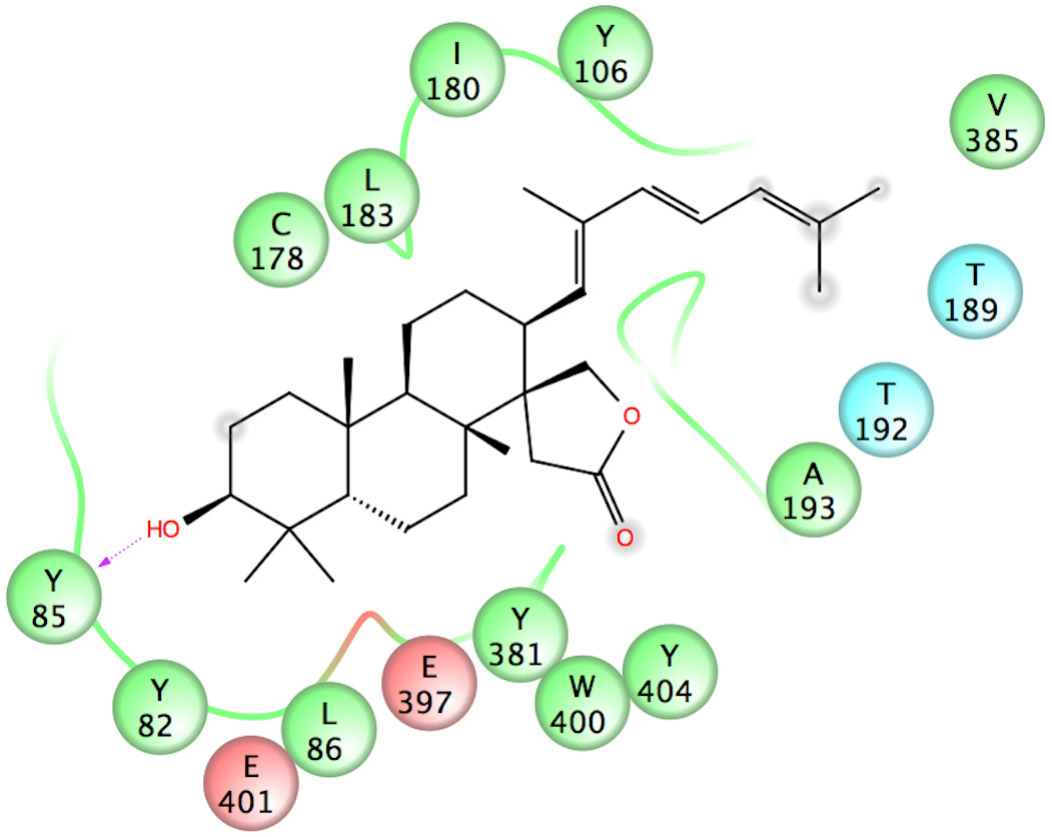
2-D interaction map of ebelin lactone in complex with the M_1_ mAChR model. Negatively-charged, polar and hydrophobic residues are depicted with red, light blue and green circles, respectively. The hydrogen bond between the OH group at position-3 and Y85 residue is indicated by a purple dashed arrow. Ebelin lactone established non-polar interactions with L183, Y82 and L86 (non-conserved residues) which are postulated to be responsible for the allosteric subtype selectivity in muscarinic receptors.

**Fig 6:**
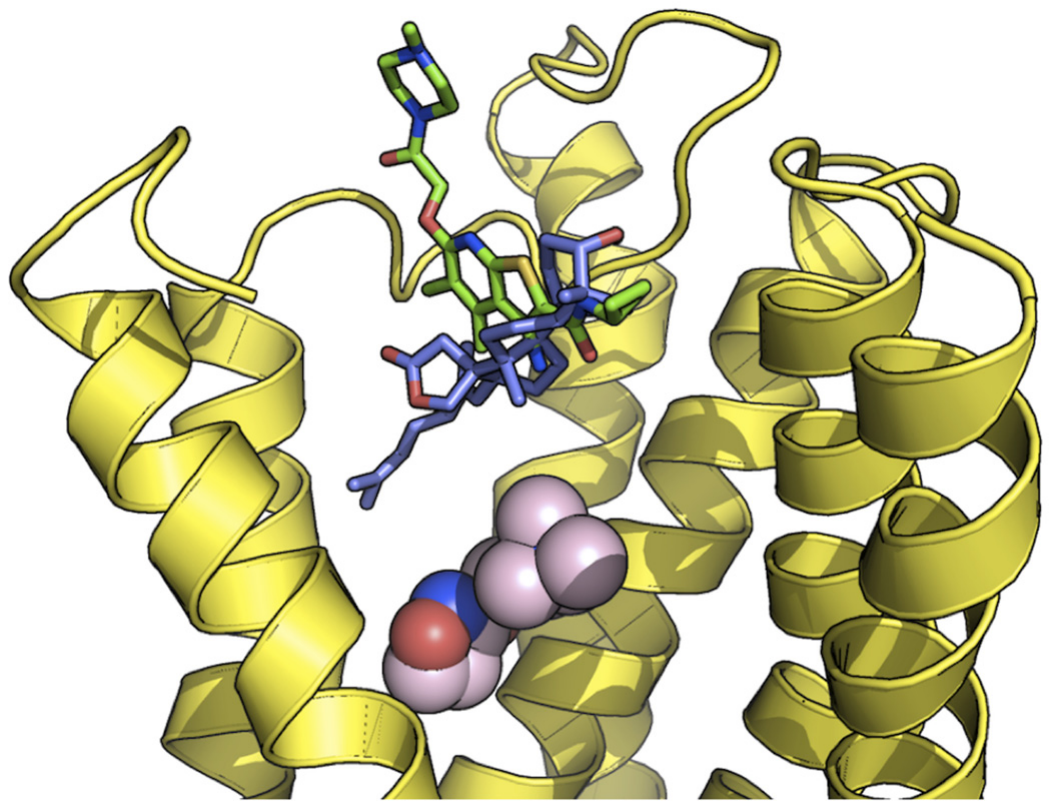
Superposition of ebelin lactone (blue) in complex with the M_1_ mAChR model and LY2119620 (green) in complex with the M_2_ mAChR. Superposition of the crystal structure of the allosteric modulator LY2119620 in complex with M_2_ mAChR (PDB code: 4MQS) with the docked ebelin lactone in M_1_ mAChR shows the overlapping binding positions of these ligands, suggesting ebelin lactone could be a M_1_ allosteric modulator. Iperoxo is shown in spheres to depict the orthosteric site of the receptor.

The investigation of the complexes obtained from the docking of ebelin lactone into M_1_ muscarinic acetylcholine receptor (mAChR) and 5-HT_2A_ models revealed that it does not fit into their primary/orthosteric binding sites (Fig 4). In the case of the M_1_ mAChR, ebelin lactone bound to a cavity directly above the orthosteric site and established interactions with a set of residues that formed the binding pocket, mainly through non-polar interactions (Figs 4 and 5). Among these interacting residues, L183 (from the extracellular loop 2), Y82 and L86 are postulated to be responsible for the muscarinic subtype selectivity. These residues are non-conserved residues among the subtypes, located above the orthosteric site that was identified by site-directed mutagenesis experiments [35–40]. It is also obvious that there is an overlapping of the ebelin lactone binding pocket with the orthosteric binding pocket, as Y106, T192, Y381, and Y404 from the orthosteric site are found within 4 Å from the bound ebelin lactone. The superposition of the recent crystal structure of M_2_ mAChR in complex with an allosteric modulator LY2119620 (PDB code: 4MQS) [41] and the docked ebelin lactone in M_1_ mAChR showed that both ligands share part of the binding cavity, suggesting that ebelin lactone could be an allosteric modulator, with a good selectivity profile (Fig 6) [42]. However, further studies are required to definitively determine the selectivity of ebelin lactone on other muscarinic subtypes.

**Fig 7:**
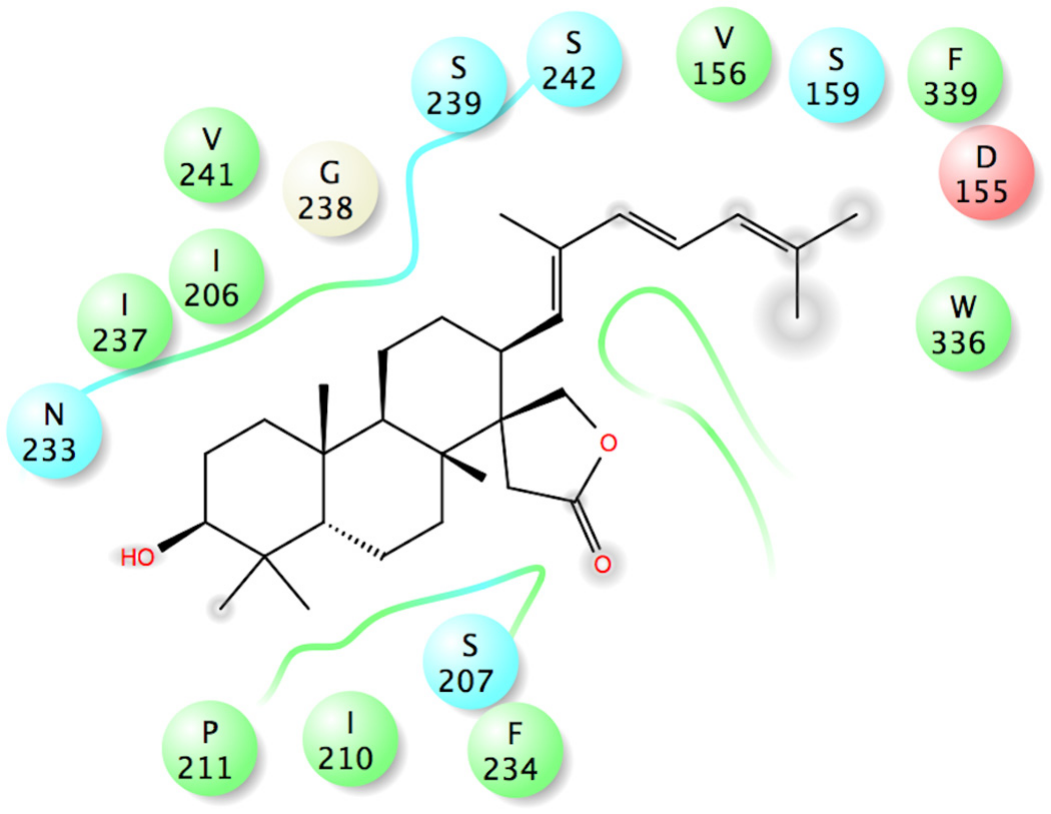
2-D interaction map of ebelin lactone in complex with the 5-HT_2A_ receptor model. Negatively-charged, polar and hydrophobic residues are depicted with red, light blue and green circles, respectively. Ebelin lactone is coordinated by a set of residues only through non-polar interactions.

**Fig 8:**
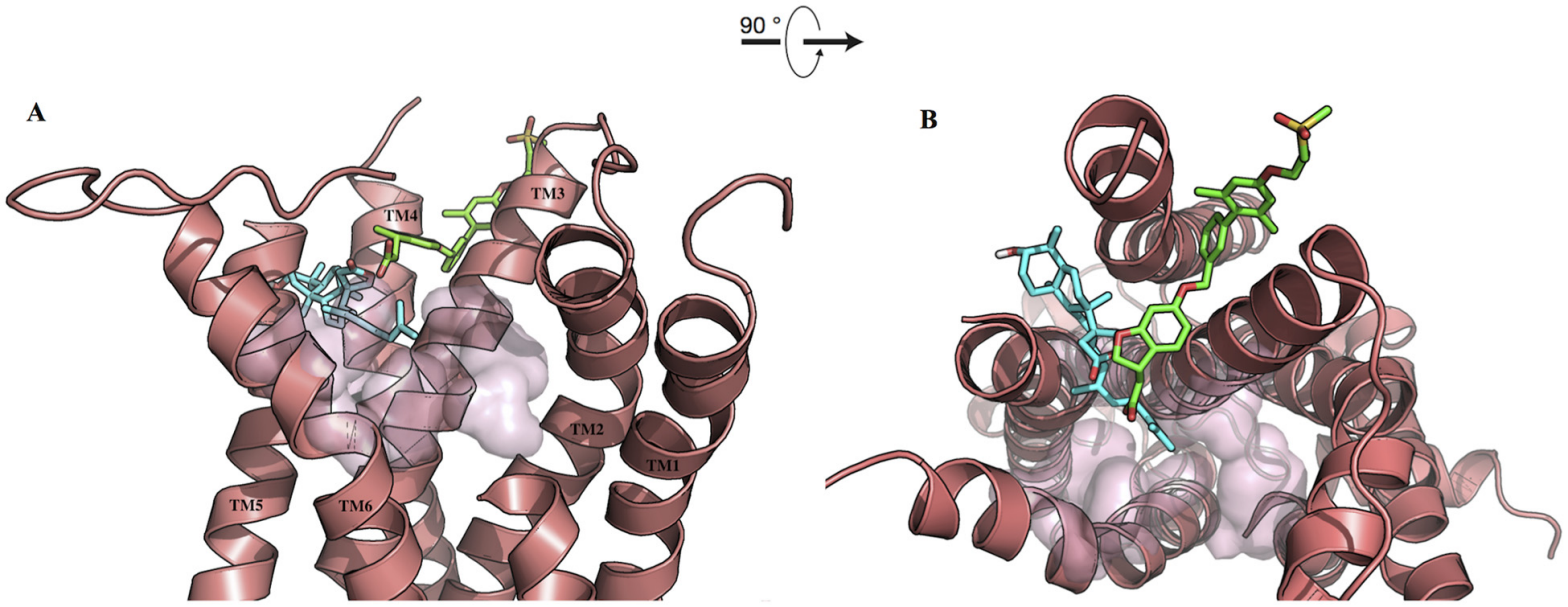
Superposition of ebelin lactone (cyan) in complex with the 5-HT_2A_ receptor and TAK-875 (green) in complex with free-fatty acid receptor 1. A. Front view and B. Top view (from the extracellular surface). Superposition of ebelin lactone in complex with the 5-HT_2A_ receptor from the docking studies and the allosteric modulator TAK-875 in complex with the free-fatty acid receptor 1 from the crystal structure (PDB code: 4PHU) shows both ligands bound to cavities in between the transmembrane helices suggesting ebelin lactone could be a 5-HT_2A_ allosteric modulator. The transparent surface represents the orthosteric site of the 5-HT_2A_ receptor. For the purpose of clarity, some of the loops and transmembrane helix are not shown.

Ebelin lactone docked to the 5-HT_2A_ receptor in a very distinct way. It did not bind to the cavity on top of the orthosteric site but, instead, almost half of the structure (the tricyclic terpenoid moiety) is found fitted in the cavity in between TM4 and TM5. The pocket seems to be an almost horizontal extension of the orthosteric binding pocket in 5-HT_2A._ Ebelin lactone is coordinated by a set of residues only through non-polar interactions, including those that are found in the orthosteric site, such as, D155, S159, S242, W336, and F339 (Fig 7) [43–47]. This unique binding of ebelin lactone is possible as the recent crystal structure of free-fatty acid receptor 1 [48] in complex with an allosteric modulator, TAK-875 (PDB code: 4PHU), revealed that the ligand binds to a non-canonical binding pocket, between TM3 and TM4 (Fig 8). Overall, binding interactions of ebelin lactone with the M_1_ and 5-HT_2A_ receptor models suggest that ebelin lactone is most likely an allosteric modulator that interacts with the residues mainly through non-polar interactions.

## Discussion

The memory enhancing and cognitive effects of *B*. *monnieri* are believed to be mediated by bacoside A, a mixture of bacoside A3, bacopacide II, bacopasaponin C and bacopaside X. However, evidence regarding the bacoside components responsible for the activity and the mechanisms of action are still unclear. This study shows that bacoside A is unlikely to be absorbed through the intestine or to penetrate the BBB, using *in silico* models. Therefore, the bacosides are likely to undergo transformation *in vivo* to remove the sugar units as well as other biotransformations, that result in metabolites that may mediate the memory enhancing and cognitive activities. This is consistent with other neuropharmacologically active plants such as ginseng [12], *Ginkgo biloba* [49] and jujube (red date) [13, 14], where their respective active constituents are formed via the metabolism of the parent compounds *in vivo*.

Unlike bacosides, the aglycones (jujubogenin and pseudo-jujubogenin) and their acid hydrolyzed derivatives (ebelin lactone and bacogenin A1) produced higher predicted binding affinity towards all the CNS receptors and stronger docking to AChE *in silico*. They also had CNS drug-like properties which suggested that they would show better oral absorption and penetration through the BBB. Among the ligands, ebelin lactone had the strongest binding of all the CNS receptors and the highest expected BBB penetration.

Although until now there are no pharmacokinetics studies on bacosides, similar studies on other saponin glycosides such as ginsenoside [50] and flavonoid glycosides such as quercetin glucoside [51] have been reported. In these studies, the parent glycosides were not found in the plasma after oral administration, while their metabolites were detected. This poor intestinal absorption of the glycosides is most likely due to their low membrane permeability. Therefore, prior to intestinal absorption into the systemic circulation, these glycosides undergo deglycosylation in the intestinal tract. In the case of ginsenoside RB1, deglycosylation of the glycosides is by gastric acid, which remove the sugar units [52]. Another proposed mechanism is the hydrolysis of glycosides by lactase phloridzin hydrolase (LPH) and cytosolic β-glucosidase (CBG). LPH, a β-glucosidase found on the outside of the brush border membrane of the small intestine, hydrolyzes the glycosides and the liberated aglycones can then be absorbed into the systemic circulation from the small intestine through passive diffusion [53]. CBG on the other hand is located intracellularly in the enterocytes and so requires active transport of the hydrophilic glycosides into the cells via the sugar transporter sodium-dependent glucose co-transporter 1 (SGLT-1). CBG is capable of hydrolyzing a broad range of glycosides including glucosides, galactosides, xylosides, arabinosides, and fucosides [54]. Besides this, glycosides that are not substrates for LPH, CBG and SGLT-1, will be transported towards the colon where they may be hydrolyzed by colonic bacteria to release the aglycones, which can then be absorbed into the systemic circulation via passive uptake [12, 14, 55].

The absorbed aglycones in the systemic circulation can then cross the BBB into the brain. This mechanism is supported by the findings that 18β-glycyrrhetinic acid, a metabolite of glycyrrhizin [56], and kaempferol and isorhamnetin [49] are detected in the brain after oral administration of the parent glycosides. In other instances, the absorbed aglycone from the intestine may go through conjugation (methylation, sulphatation and glucuronidation) and exist in the plasma in the conjugated forms as with flavonoid aglycones [57]. Flavonoids in the form of aglycones and conjugated forms are able cross the BBB. During passage of the BBB, the conjugates may be metabolized back to the parent aglycone, which then enters the central nervous system [58]. Therefore, similar to glycosides from other CNS active plants and our *in silico* results here a similar pharmacokinetics behavior is expected for the bacosides.

The findings from the *in vitro* radioligand receptor binding assays confirmed the favorable affinity of the aglycone derivative, ebelin lactone towards M_1_ (*K*_i_ = 0.45 μM) and 5-HT_2A_ (4.21 μM) receptors, where the binding activities are similar to other known M_1_ and 5-HT agonists. In contrast, the aglycone jujubogenin did not give significant binding affinity towards the receptors. This difference could be explained by the presence of the carbonyl oxygen of the lactone ring in ebelin lactone, which is lacking in jujubogenin. The carbonyl oxygen of a lactone ring has previously been reported to be essential for the activity of pilocarpine at M_1_ receptors [59]. In the current work, the *in silico* studies were unable to identify the precise role of the carbonyl oxygen in the binding of ebelin lactone, perhaps due to limitations in the docking and scoring functions used, such as not allowing full conformational flexibility in the receptor. However, they did suggest that ebelin lactone could act as an allosteric modulator via non-polar interactions. Such allosteric binding of the aglycone derivatives to the M_1_ and 5-HT_2A_ receptors, distinct from orthosteric interaction, may offer greater selectivity and reduced side effects, and may conceivably contribute to the cognitive function and safety of *B*. *monnieri* [60–63].

M_1_ mAChR and serotonin 5-HT_2A_ receptors are expressed abundantly in brain regions essential for cognitive functions such as the prefrontal cortex and hippocampus. The stimulation of these receptors by their respective agonists has been shown to improve cognition and to enhance learning in humans and animal models [33, 64]. M_1_ receptors are associated with cholinergic transmission whereas 5-HT_2A_ receptors are associated with both cholinergic and glutamatergic transmission, and are implicated in cognition by regulating the release of these and other neurotransmitters [32, 33]. Cognitive functions are said to be dependent on the ability of neurons to change their function i.e. neural plasticity [65]. The pyramidal neurons (pyramidal cells) are the primary excitation units in the mammalian cortical structures which play important roles in cognition through their neural plasticity (synaptic plasticity) function and are also expressed abundantly in the prefrontal cortex and hippocampus [66]. At cellular levels the M_1_ receptors are located on the dendrites of cortical pyramidal cells [67, 68] whereas the 5-HT_2A_ receptors are located on both the dendrites of cortical pyramidal cells and the interneurons [69]. Activation of M_1_ and 5-HT_2A_ metabotrobic receptors in pyramidal cells activates phospholipase C (PLC), which subsequently promotes the release of diacylglycerol (DAG) and inositol triphosphate (IP3), stimulate protein kinase C (PKC) activity and Ca^2+^ release, leading to activations of signal transduction pathways that result in increased neural plasticity [70, 71]. In addition, the location of 5-HT_2A_ receptors in the cortex and hippocampus on cholinergic [72] and glutamatergic [73] axon terminals serves to regulate the release of these transmitters where the increased release of acetylcholine and glutamate are expected to enhance learning [33]. Pyramidal cells use glutamate as their excitatory neurotransmitter, and GABA as their inhibitory neurotransmitter [74].

There is evidence that the mechanisms of action of *B*. *monnieri* could be attributed to a combination of cholinergic modulation especially through the muscarinic cholinergic receptor. *B*. *monnieri* extract has been reported to alleviate the amnesic effects of scopolamine, a muscarinic receptor antagonist, suggesting a crucial role of muscarinic receptors in the action of *B*. *monnieri* [15, 75]. Furthermore, the administration of *B*. *monnieri* for two weeks reversed the depletion of acetylcholine, reduced choline acetylase activity and decreased muscarinic cholinergic receptor binding in the frontal cortex and hippocampus of rats with AD, induced by the neurotoxin colchicine [76]. In addition to this, *B*. *monnieri* extract was found to induce neurite and neuronal dendritic growth [77, 78], and studies have shown that muscarinic receptor activation plays a key role in neurite outgrowth [79, 80]. Previous work has demonstrated that treatment with *B*. *monnieri* extract caused an increase in 5-HT levels in the hippocampus, hypothalamus and cerebral cortex of rats [8, 81]. Charles et al. [82] also found that *B*. *monnieri* extract caused a significant up-regulation in the synthesis of 5-HT and altered the ACh level, and proposed that the elevated 5-HT level may activate their receptor to facilitate the release of ACh and thus enhance learning ability and memory.

*In vitro* studies suggest *B*. *monnieri* extract did not inhibit AChE directly [15, 83]. However, brain homogenate obtained from rats fed with *B*. *monnieri* extract showed anti-AChE activity [15, 84]. Treatment with bacosides on aged rats for a 3 months period appeared to enhance the synthesis and availability of acetylcholine rather than affecting the activity of AChE [85]. Our *in silico* findings suggest that the aglycones dock on the catalytic site of AChE better than the parent bacosides. However, the same pattern is not reflected in the *in vitro* AChE inhibition study. The reason for the discrepancy has not been determined. It is conceivable that the crystal structure of AChE for this study optimized for fasciculin and donepezil is not suitable for bacosides and their aglycones as their structures are quite different. It is also worth while noting that pseudojujubogenin and bacogenin A1, which have not been tested, may bind to AChE and cause inhibition.

Our findings are based on bacoside A (bacoside A3, bacopaside X, bacopaside II, bacopasaponin C), its aglycones (jujubogenenin and pseudojujubogenin) and its derivatives (ebelin lactone and bacogenin A1). Although both pseudojujubogenin and bacogenin A1 were evaluated by *in silico* studies, they were not evaluated with *in vitro* receptor binding assays and the AChE inhibition assay. It is conceivable that they may interact with the CNS receptors and bind to the catalytic site of AChE to contribute to the cognitive effect of *B*. *monnieri*.

This study used validated *in silico* receptor and AChE models from our previous work to predict the activities of bacosides, aglycones and their derivatives. However, the adopted *in silico* models were built and validated using ligands structurally different from that of the bacosides tested. Therefore, the models may not be fully reliable for bacosides. Nevertheless, this combination of *in silico* and *in vitro* studies gives an overall picture of absorption (pharmacokinetics) and pharmacodynamics of bacosides.

## Conclusions

In this study, we have demonstrated through a combination of *in silico* and *in vitro* experiments that the bacoside aglycone derivative, ebelin lactone, has better CNS drug-like and receptor-binding properties compared to the parent compound. Hence, we suggest *B*. *monnieri* constituents may be transformed and metabolized to the active form *in vivo* before exerting their pharmacological activity. Additional studies are required to determine the actual metabolites of *B*. *monnieri* in order to further elucidate the memory enhancing and cognitive actions of this plant. Ebelin lactone may also be an interesting CNS drug candidate that is worthy of further investigation. The results from such studies will give more of an indication of the potential of *B*. *monnieri* for the treatment of AD.
